# Differences in Expansion Potential of Naive Chimeric Antigen Receptor T Cells from Healthy Donors and Untreated Chronic Lymphocytic Leukemia Patients

**DOI:** 10.3389/fimmu.2017.01956

**Published:** 2018-01-10

**Authors:** Jean-Marc Hoffmann, Maria-Luisa Schubert, Lei Wang, Angela Hückelhoven, Leopold Sellner, Sophia Stock, Anita Schmitt, Christian Kleist, Ulrike Gern, Angelica Loskog, Patrick Wuchter, Susanne Hofmann, Anthony D. Ho, Carsten Müller-Tidow, Peter Dreger, Michael Schmitt

**Affiliations:** ^1^Cellular Immunotherapy, GMP Core Facility, Department of Internal Medicine V, Heidelberg University Hospital, Heidelberg, Germany; ^2^National Center for Tumor Diseases (NCT), Heidelberg, Germany; ^3^Department of Nuclear Medicine, Heidelberg University Hospital, Heidelberg, Germany; ^4^Department of Immunology, Genetics and Pathology, Science for Life Laboratory, Uppsala University, Uppsala, Sweden; ^5^Medical Faculty Mannheim, Institute of Transfusion Medicine and Immunology, Heidelberg University, German Red Cross Blood Service Baden-Württemberg – Hessen, Mannheim, Germany

**Keywords:** chimeric antigen receptor, immunotherapy, CD19, T cell subpopulations, naive T cells, cytokines, chronic lymphocytic leukemia, T cell expansion

## Abstract

**Introduction:**

Therapy with chimeric antigen receptor T (CART) cells for hematological malignancies has shown promising results. Effectiveness of CART cells may depend on the ratio of naive (T_N_) vs. effector (T_E_) T cells, T_N_ cells being responsible for an enduring antitumor activity through maturation. Therefore, we investigated factors influencing the T_N_/T_E_ ratio of CART cells.

**Materials and methods:**

CART cells were generated upon transduction of peripheral blood mononuclear cells with a CD19.CAR-CD28-CD137zeta third generation retroviral vector under two different stimulating culture conditions: anti-CD3/anti-CD28 antibodies adding either interleukin (IL)-7/IL-15 or IL-2. CART cells were maintained in culture for 20 days. We evaluated 24 healthy donors (HDs) and 11 patients with chronic lymphocytic leukemia (CLL) for the composition of cell subsets and produced CART cells. Phenotype and functionality were tested using flow cytometry and chromium release assays.

**Results:**

IL-7/IL-15 preferentially induced differentiation into T_N_, stem cell memory (T_SCM_: naive CD27+ CD95+), CD4+ and CXCR3+ CART cells, while IL-2 increased effector memory (T_EM_), CD56+ and CD4+ T regulatory (T_Reg_) CART cells. The net amplification of different CART subpopulations derived from HDs and untreated CLL patients was compared. Particularly the expansion of CD4+ CART_N_ cells differed significantly between the two groups. For HDs, this subtype expanded >60-fold, whereas CD4+ CART_N_ cells of untreated CLL patients expanded less than 10-fold. Expression of exhaustion marker programmed cell death 1 on CART_N_ cells on day 10 of culture was significantly higher in patient samples compared to HD samples. As the percentage of malignant B cells was expectedly higher within patient samples, an excessive amount of B cells during culture could account for the reduced expansion potential of CART_N_ cells in untreated CLL patients. Final T_N_/T_E_ ratio stayed <0.3 despite stimulation condition for patients, whereas this ratio was >2 in samples from HDs stimulated with IL-7/IL-15, thus demonstrating efficient CART_N_ expansion.

**Conclusion:**

Untreated CLL patients might constitute a challenge for long-lasting CART effects *in vivo* since only a low number of T_N_ among the CART product could be generated. Depletion of malignant B cells before starting CART production might be considered to increase the T_N_/T_E_ ratio within the CART product.

## Introduction

The advent of T cells expressing chimeric antigen receptor T (CART) cells for the treatment of cancer patients represents a milestone in the field of immunotherapy (1). Various clinical trials have been undertaken in recent years, especially for relapsed or refractory CD19+ hematologic malignancies such as acute lymphoblastic leukemia, chronic lymphocytic leukemia (CLL), and non-Hodgkin lymphoma (2–7). For clinical CART application, reproducibility and safe generation of CART cells is of crucial relevance. T cells from patients (autologous) or donors (allogeneic) are genetically modified *via* viral or non-viral vectors to express a recombinant transmembrane receptor on the cell surface. The so-called chimeric antigen receptor (CAR) recognizes CD19+ malignant B cells with the extracellular antibody-derived and antigen-specific recognition domain (single chain variable fragment) (8, 9). The cytoplasmic signaling domain is constituted of a CD3zeta stimulatory domain combined to costimulatory signaling components such as CD28 (10, 11), CD137/4−1BB (12), or OX40, either alone for so-called second generation or in combination for third generation CARs (13). However, while some patients have displayed long-lasting CART responses (14), expansion and particularly persistence of CART cells in other patients have lasted only for few weeks (5, 15). Since clinical response correlates with long-term detection of the engineered T cells (16), short-term CART cells are limited in their capacity to fully eradicate cancer cells (17). The phenotype of T cells administered to patients often correlates with antitumor reactivity *in vivo* (18): Particularly, less-differentiated naive (T_N_) and central memory (T_CM_) T cells in contrast to the more differentiated effector memory (T_EM_) and effector (T_E_) T cells seems to play an essential role in CART persistence (19–21). Effectiveness of CART cells might therefore depend on the proportion of naive vs. effector cells (T_N_/T_E_ ratio) in the final CART product. It still remains to be elucidated why for some patients a high proportion of naive cells within their CART product can be expanded, whereas for others efficient expansion of this subtype could not be achieved despite optimal culture conditions.

Hence, we monitored the evolution and amplification of CART subpopulations (T_N_, T_CM_, T_EM_, and T_E_) and particularly the T_N_/T_E_ ratio derived from both healthy donors (HDs) and untreated CLL patients during CART culture. For CART generation, the most commonly used cytokine stimulation cocktails are either interleukin (IL)-7/IL-15 (22–25) or IL-2 (5). In order to assess a specific influence of those stimulating cytokines on the net amplification of CART subtypes, CART cells were generated simultaneously under both conditions. The starting cell material, i.e., peripheral blood mononuclear cells (PBMCs), from HDs as well as CLL patients was screened to analyze whether a correlation exists between the phenotypic composition of PBMCs and the subsequent expansion of highly effective, long-lasting naive CART cells during culture. We demonstrate that the expansion of naive T cells is clearly associated with the specific cellular composition of PBMCs used for CART generation and identify factors determining optimal generation of clinical CART products.

## Materials and Methods

### CD19.CART Generation and Culture

Cryopreserved human PBMCs from HDs (from the blood bank Mannheim, DRK-Blutspendedienst Baden-Württemberg-Hessen) and untreated CLL patients (from Heidelberg University Hospital, protocol number: S-254/2016) were thawed and activated with anti-CD3/anti-CD28 antibodies (Biozol, Eching, Germany) at a concentration of 5 × 10^5^ cells/ml culture medium. For activation of PBMCs, non-tissue culture-treated 24-well plates (Corning, Wiesbaden, Germany) had been previously coated over night with 0.5 ml of 1 µg/ml anti-CD3 and 1 µg/ml anti-CD28, diluted in Aqua ad iniect (Fresenius, Bad Homburg, Germany). Culture medium consisted of 50% RPMI 1640 (Thermo Fisher Scientific, Waltham, MA, USA) and 50% Click’s Medium (EHAA) (Irvine Scientific, Santa Ana, CA, USA), with 10% heat-inactivated fetal bovine serum (FBS) (Thermo Fisher Scientific) and 2 mM l-glutamine (Thermo Fisher Scientific). Two different cytokine cocktails were added on day 2 of culture: IL-7/IL-15 (R&D Systems, Minneapolis, MN, USA) vs. IL-2 (Novartis, Nuremberg, Germany), at concentrations according to our GMP protocol (IL-7: 4.4 × 10^3^ U/ml, IL-15: 100 U/ml, IL-2: 100 U/ml). On day 3, activated T cells (5 × 10^5^ cells in 0.5 ml + 1.5 ml retroviral supernatant per well) were transduced with a CD19.CAR-CD28-CD137zeta third generation retroviral vector (kindly provided by Dr. Malcolm Brenner at the Center for Cell and Gene Therapy, Baylor College of Medicine, Houston, TX, USA) in a 24-well non-tissue culture-treated plate, previously coated with 7 µg/ml RetroNectin (Takara Bio, Shiga, Japan) in Dulbecco’s phosphate-buffered saline (PBS) (Sigma-Aldrich, Taufkirchen, Germany). Viability of CART cells was assessed through Trypan blue staining. Medium change with fresh addition of cytokines was performed on days 3, 7, 10, 14, and 17 (1 × 10^6^ cells/ml). CART cells were cultivated in 6-well tissue culture plates (Sarstedt, Nümbrecht, Germany) and transferred to T75 tissue culture flasks (Sarstedt) when the total cell number reached 15 × 10^6^ cells. On days 14–17, an aliquot of CART cells was cryopreserved in FBS + 10% DMSO (Sigma-Aldrich). Total culture period lasted 20 days. The net amplification of CART cells and specific subpopulations from day 7 up to day 20 was calculated using the following formula: net amplification = absolute cell number × % CD3+ CART × % specific subpopulation.

### Cell Lines

CD19+ Burkitt lymphoma (Daudi) cells (Leibniz Institute DSMZ—German Collection of Microorganisms and Cell Cultures, Braunschweig, Germany) were expanded in cell culture medium (RPMI 1640 supplemented with 10% FBS and 2 mM l-glutamine) in T75 tissue culture flasks (Sarstedt) at 37°C and 5% CO_2_ in a humidified incubator (Memmert, Schwabach, Germany) and split 1:3 every three days. Cells were used as target cells for the cytotoxicity and cytokine expression assays.

### Flow Cytometry

Flow cytometric analyses were performed on days 0, 7, 10, 14, 15, 17, and 20 of CART culture. Dead cells were excluded using the LIVE/DEAD^®^ Fixable Near-IR Dead Cell Stain Kit (Thermo Fisher Scientific). Surface marker staining was performed in order to assess the corresponding subpopulation markers on T_N_ (CD45RA+ CCR7+), T_CM_ (CD45RA− CCR7+), T_EM_ (CD45RA− CCR7−), and T_E_ (CD45RA+ CCR7−) cells. The following fluorochrome-conjugated antibodies were used: CD45RA-APC, CD8-PerCP, CD8-Pacific Blue, CD95-PE-Cy5, programmed cell death 1 (PD-1)-Alexa Fluor 488, Tim-3-Brilliant Violet 421, CXCR3-Alexa Fluor 488, CD56-Alexa Fluor 488, γ/δ TCR-Brilliant Violet 421, CD25-PE-Cy7, CD20-APC-Cy7 (all from Biolegend, San Diego, CA, USA), CCR7-PE-Cy7, CD3-PE eFluor 610, CD4-Alexa Fluor 700, HLA-DR-PerCP, CD62L-eFluor 450 (all from eBioscience, San Diego, CA, USA), CD3-V500, CD27-FITC, CD45-FITC, CD19-APC, and CD34-PE (all from BD Biosciences, Franklin Lakes, NJ, USA). CART cells were stained with an anti-human goat F(ab)_2_ IgG (H+L)-PE antibody (Dianova, Hamburg, Germany). As the F(ab)_2_ IgG (H+L) antibody recognizes IgG molecules, present on both the CAR receptor and B cells, the following gating strategy was applied: first gate assessed viable cells, second gate CD3+ cells and third gate CD3+ CART cells (Figure S1 in Supplementary Material). Surface marker staining was performed for 30 min at room temperature. Intracellular staining was used for detection of expression of TNF-α (BV421, BD Biosciences), IFN-γ (Alexa Fluor 488, Biolegend), and IL-2 (BV510, BD Biosciences) by CART cells after coculturing with CD19+ Daudi cells for 6 h. Brefeldin A was used for blocking cytokine secretion. The percentage of FoxP3+ T_Reg_ cells after stimulation with IL-7/IL-15 vs. IL-2 was measured using the FoxP3 Staining Buffer Set (Miltenyi Biotec, Bergisch-Gladbach, Germany). After staining, cells were fixed with PBS + 1% paraformaldehyde + 3 mM ethylene-diamine-tetra-acetic acid. All acquisitions of data were performed on an LSRII device (BD Biosciences) and data were analyzed using FlowJo software (FlowJo, Ashland, OR, USA).

### Chromium-51 Release Assay/Cytotoxicity Assay

Cytotoxic efficacy of the manufactured CART cells was assessed by a standard chromium (Cr-51) release assay as previously described (26). CD19+ Burkitt lymphoma (Daudi) cells (DSMZ, Braunschweig, Germany) (target cells) were labeled for 2 h with Cr-51 (Hartmann Analytic, Braunschweig, Germany) and coincubated for 4 h with CART cells (effector cells) in 96-well U-bottom microplates (Greiner Bio-One, Frickenhausen, Germany). Maximum Cr-51 release of target cells was measured following complete lysis by adding PBS + 1% Triton X-100 (Sigma-Aldrich), whereas background was assessed *via* spontaneous release of Cr-51 of target cells kept in culture medium without the addition of CART cells. Effector to target cell ratios ranged from 10:1 to 1:1. Cr-51 release was measured in a liquid scintillation counter (PerkinElmer, Waltham, MA, USA) after adding Ultima Gold liquid scintillation cocktail (PerkinElmer) to a culture supernatant aliquot. Experiments were performed in triplicates. Cytotoxicity was calculated as percentage of specific lysis according to the following formula: % specific lysis = (Cr-51 release in the test well − spontaneous Cr-51 release)/(maximum Cr-51 release − spontaneous Cr-51 release) × 100.

### Statistical Analysis

Statistical analyses were performed using SPSS software (IBM, Armonk, NY, USA). Equal variances between values from HDs vs. untreated CLL patients were determined by an F-test and subsequent *p*-values were calculated using the two-tailed independent samples t-test. In order to compare the influence of the cytokine cocktails IL-7/IL-15 vs. IL-2, a paired samples t-test was used. Graphs were designed by Excel (Microsoft, Redmond, WA, USA) and Keynote (Apple, Cupertino, CA, USA). If not otherwise mentioned, results are displayed as mean ± SD. Correction for multiple comparisons was performed using the Holm-Bonferroni method.

## Results

### Screening of HDs and Patients

The cellular composition of cryopreserved human PBMCs for CART production from 24 HDs and 11 untreated CLL patients was analyzed: the percentage of B cells as well as CD3+ T cells within lymphocytes, the proportions of CD4+ and CD8+ cells and the CD4:CD8 ratio were measured. Moreover, the CD4+ naive, CD8+ naive and the total naive cells within the CD3+ fraction as well as the absolute naive cell number were determined (Table 1). Within the cells of HDs, CD4:CD8 ratios were heterogeneously distributed ranging from 0.4 to 5.3. In 8 out of 24 (33%) HDs the CD4:CD8 ratio was <1, i.e., more CD8+ than CD4+ cells were found within the peripheral blood. The CD3+ cell proportion within total PBMCs ranged from 41.5 to 90.0% (mean ± SD: 73 ± 11%). The percentage of naive cells within the CD3+ fraction ranged from 10.2 to 39.4% (mean ± SD: 24 ± 7%) and did not correlate with the percentage of CD3+ cells or with the CD4:CD8 ratio.

**Table 1:** Screening of HDs and CLL patients (P) for lymphocyte subsets within PBMCs.

A healthy donors									

*n* = 24	Subsets within lymphocytes (%)								Absolute number naive CD3+ T cells (per 100 lymphocytes)
	B cells	CD3+	% total CD3+ T cells						
			CD4+	CD8+	CD4:CD8 ratio	CD4+ naive	CD8+ naive	Total naive	
HD 1	11.4	82.0	66.9	29.9	2.2	30.0	9.2	39.4	32.3
HD 2	17.4	77.3	44.6	44.5	1.0	21.7	14.2	36.4	28.1
HD 3	19.7	76.2	31.2	55.2	0.6	6.9	26.7	34.1	26.0
HD 4	10.1	76.6	61.2	35.5	1.7	20.0	10.3	30.5	23.4
HD 5	11.0	75.3	49.6	45.6	1.1	15.0	14.7	30.0	22.6
HD 6	8.1	82.1	57.5	37.7	1.5	13.7	12.3	26.2	21.5
HD 7	26.6	64.1	43.1	50.2	0.8	10.0	23.0	33.4	21.4
HD 8	15.5	80.5	35.6	59.8	0.6	7.8	17.3	25.3	20.4
HD 9	8.8	86.4	81.1	15.2	5.3	20.4	2.9	23.4	20.2
HD 10	8.1	82.4	54.3	36.7	1.5	17.4	5.8	23.4	19.3
HD 11	20.0	74.6	48.0	45.5	1.1	10.5	14.7	25.5	19.0
HD 12	16.5	78.3	55.7	42.3	1.3	18.9	5.1	24.2	18.9
HD 13	19.1	66.1	46.7	49.3	0.9	12.5	15.2	27.9	18.4
HD 14	22.8	69.3	54.8	41.5	1.3	17.0	8.7	26.0	18.0
HD 15	9.3	90.0	36.6	55.0	0.7	8.8	10.3	19.5	17.6
HD 16	20.5	64.1	69.4	28.0	2.5	18.6	4.5	23.2	14.9
HD 17	36.3	58.0	43.8	45.8	1.0	10.4	11.7	22.6	13.1
HD 18	17.8	72.4	62.8	33.7	1.9	12.3	5.5	17.8	12.9
HD 19	20.2	58.4	49.4	38.0	1.3	15.5	5.8	21.5	12.6
HD 20	24.1	76.0	28.0	65.0	0.4	5.8	10.5	16.5	12.5
HD 21	15.8	64.2	48.4	42.4	1.1	14.2	4.9	19.2	12.3
HD 22	17.4	74.2	44.9	51.2	0.9	10.6	2.5	13.3	9.9
HD 23	16.2	74.4	48.3	48.3	1.0	5.6	4.4	10.2	7.6
HD 24	54.5	41.5	43.6	51.3	0.9	8.3	9.3	18.1	7.5

**B CLL patients**									

***n* = 11**									

P 1	92.8	6.9	51.7	40.1	1.3	13.6	6.3	22.0	1.5
P 2	96.1	3.7	55.2	38.4	1.4	24.8	11.5	37.5	1.4
P 3	92.0	6.3	64.5	26.0	2.5	17.0	1.5	19.2	1.2
P 4	71.5	23.8	32.3	65.6	0.5	0.3	3.5	3.9	0.9
P 5	94.7	2.3	57.1	31.5	1.8	22.3	5.2	29.8	0.7
P 6	92.5	5.5	42.4	48.2	0.9	6.7	5.0	13.1	0.7
P 7	93.8	5.6	41.0	56.0	0.7	9.0	2.8	12.8	0.7
P 8	95.3	3.6	55.6	32.8	1.7	13.7	1.7	17.3	0.6
P 9	92.5	2.0	60.2	31.8	1.9	18.4	3.7	24.8	0.5
P 10	80.6	11.9	44.0	51.1	0.9	1.4	2.5	4.6	0.5
P 11	95.9	2.6	29.3	64.4	0.5	2.3	2.3	5.7	0.1

*Cryopreserved PBMCs from HDs (*n* = 24) (**A**) and untreated CLL patients (*n* = 11) (**B**) were analyzed. The number of B cells as well as CD3+, CD4+, and CD8+ T cells, the CD4:CD8 ratio, CD4+ and CD8+ naive T cells and the absolute number of naive CD3+ T cells per 100 lymphocytes are listed. Data from HDs and patients with chronic lymphocytic leukemia (CLL) are shown from highest to lowest frequency of naive T cells (last column)*.

Within cells of patients, CD4:CD8 ratios were also heterogeneously distributed ranging from 0.5 to 2.5. In 5 out of 11 (45%) patients the CD4:CD8 ratio was <1. Given that analyzed patients were untreated CLL patients containing high percentages of B cells within their PBMCs, the CD3+ cell proportion was significantly lower compared to HDs (*p* = 2.35E-19) and ranged from 2.0 to 23.8% (mean ± SD: 7 ± 6%). The percentage of naive cells within the CD3+ fraction ranged from 3.9 to 37.5% (mean ± SD: 17 ± 11%) and was significantly lower in comparison to HDs (*p* = 0.026).

Following this screening for subpopulations within the PBMCs, we selected individuals from both HDs (HD 7, HD 10, HD 21; *n* = 3) and untreated CLL patients (P 2, P 4, P 5; *n* = 3) for the production of CART cells.

### Generation of CART Cells from HDs and Patients

Following screening, CART cells were generated from six different donors with comparable starting frequencies of T_N_, i.e., three HDs and three untreated CLL patients. Thawed cryopreserved PBMCs were stimulated with anti-CD3/anti-CD28 antibodies plus either IL-7/IL-15 or IL-2 and cultured *ex vivo* for up to 20 days. The evolution of specific CART subpopulations over time was assessed by phenotypic analyses on days 0, 7, 10, 14, 17, and 20. Transduction efficiency on day 7 of culture was comparable and averaged 54 ± 32% and 52 ± 32% for IL-7/IL-15 and IL-2 cultures, respectively.

**Figure 1:**
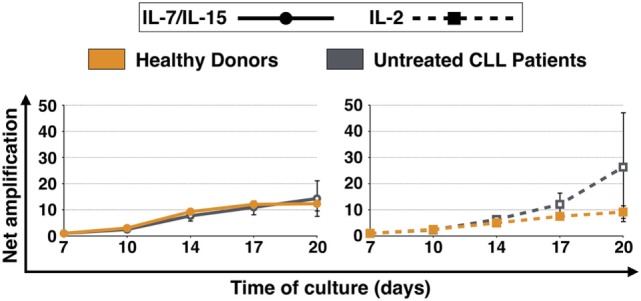
Net amplification of all chimeric antigen receptor T (CART) cells during culture. CART cells were generated from cryopreserved peripheral blood mononuclear cells of three healthy donors (*n* = 3, orange lines) and three untreated chronic lymphocytic leukemia patients (*n* = 3, gray lines) after activation of T cells with anti-CD3/anti-CD28 antibodies (days 0–3), transduction with a CD19.CAR-CD28-CD137zeta retroviral vector (day 3), and culture with either interleukin (IL)-7/IL-15 (*n* = 3, solid lines) or IL-2 (*n* = 3, hatched lines) (days 2–20). The net amplification of all CD3+ CART cells were determined by flow cytometry for each treatment condition and time point as indicated. Mean values of all twelve cell samples ± SDs are plotted.

The net amplification factor of CART cells takes into account the absolute cell number, the percentage of CD3+ cells as well as the transduction efficiency from day 7 until day 20 of culture and was used to assess the expansion rate of CART cells throughout culture. Figure 1 displays the expansion levels of CART cells for both HDs and untreated CLL patients after stimulation with IL-7/IL-15 or IL-2. When stimulated with IL-7/IL-15, the expansion rate of CART cells from HDs and patients showed no significant difference [net amplification on final culture day 20 (IL-7/IL-15): 12-fold (HDs) and 14-fold (CLL patients)]. When stimulated with IL-2, however, CART cells of patients showed a higher expansion rate compared to CART cells of HDs [on final culture day 20 (IL-2): 26-fold expansion (CLL patients) vs. 9-fold expansion (HDs)].

**Figure 2:**
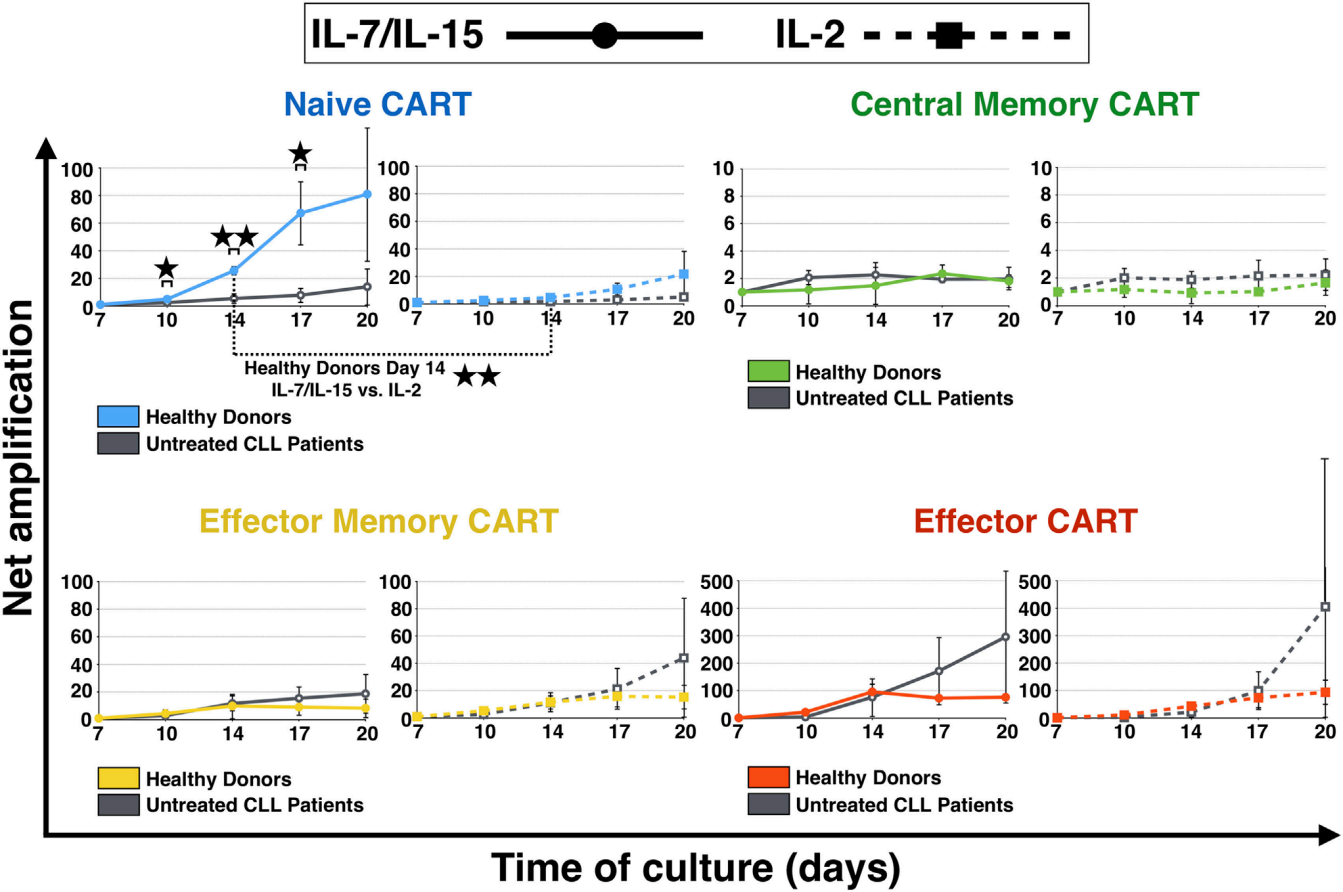
Net amplification of chimeric antigen receptor T (CART) subpopulations during culture. The net amplification of T_N_ (CD45RA+ CCR7+), T_CM_ (CD45RA − CCR7+), T_EM_ (CD45RA − CCR7−), and T_E_ (CD45RA+ CCR7−) CART cells from healthy donors (*n* = 3, colored lines) and untreated chronic lymphocytic leukemia patients (*n* = 3, gray lines) was determined. The cellular subsets were also compared according to the mode of stimulation, either with interleukin (IL)-7/IL-15 (*n* = 3, solid lines) or with IL-2 (*n* = 3, hashed lines). Mean values of the net amplifications of the various cellular subsets were calculated for each group and are represented with SD. Significance is represented as * for *p* < 0.05 and ** for *p* < 0.01. Exact data are specified in the text.

Subsequently, expansion of different subpopulations of CART cells during culture, i.e., T_N_, T_CM_, T_EM_, and T_E_, was investigated (Figure 2). The naive CART cells stimulated with IL-7/IL-15 were the subpopulation that showed the most pronounced differences when HDs and patients were compared, showing statistical significance on days 10, 14, and 17. While CART_N_ cells from HDs expanded notably between day 7 and day 20 (expansion level day 10: 5-fold, day 14: 25-fold, day 17: 67-fold, day 20: 81-fold), CART_N_ cells from untreated CLL patients showed a reduced capacity to expand [expansion level day 10: 2-fold (*p* = 0.019), day 14: 5-fold (*p* = 0.002), day 17: 8-fold (*p* = 0.012), day 20: 14-fold]. When stimulated with IL-2, expansion levels of CART_N_ cells were lower compared to IL-7/IL-15 stimulation, e.g., on day 14: 25-fold expansion (IL-7/IL-15) vs. 5-fold expansion (IL-2) (*p* = 0.004).

The most stable subpopulation during culture was the central memory subset. T_CM_ showed no difference of expansion when stimulated with the two different cytokine stimulation cocktails (day 20: twofold expansion for both IL-7/IL-15 and IL-2 stimulation).

Until day 14, also T_EM_ CART cells displayed similar expansion patterns independent of the cytokine cocktail used. After day 14, however, T_EM_ derived from patients showed higher proliferation levels compared to HDs [highest effect with IL-2: 44-fold expansion (patients) vs. 15-fold expansion (HDs)].

Finally, T_E_ cells were the subpopulation of CART cells that expanded the most during culture: the amplification factor for T_E_ was above 75 for HDs and the strongest proliferation was detected for untreated CLL patients [day 20: 296-fold expansion (IL-7/IL-15), 405-fold expansion (IL-2)].

**Figure 3:**
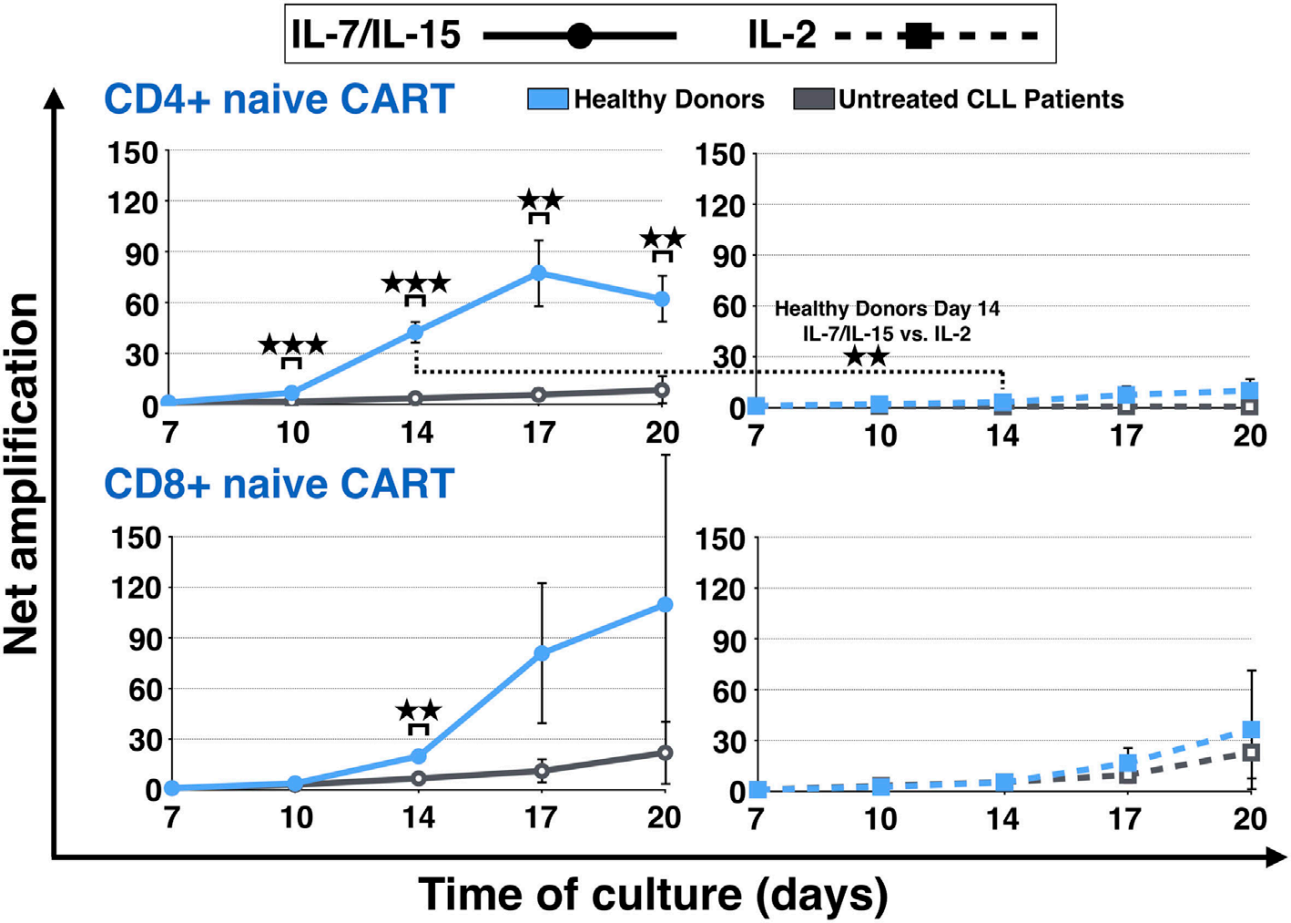
Net amplification of CD4+ and CD8+ naive chimeric antigen receptor T (CART) cells during culture. The net amplification of the CD4+ and CD8+ naive CART subtypes from healthy donors (*n* = 3, blue lines) and untreated chronic lymphocytic leukemia patients (*n* = 3, gray lines) was determined. The cellular subsets were also compared according to the mode of stimulation, either with interleukin (IL)-7/IL-15 (*n* = 3, solid lines) or with IL-2 (*n* = 3, hashed lines). Mean values of the net amplifications of the various cellular subsets were calculated for each group and are represented with SD. Significance is represented as ** for *p* < 0.01 and *** for *p* < 0.001. Exact data are specified in the text.

The expansion of the naive CART subpopulation was further evaluated by analyzing the evolution of the CD4+ and CD8+ fractions of HDs and untreated CLL patients (Figure 3). For both CD4+ and CD8+ CART_N_ cells, a more pronounced expansion during culture was detected when they were derived from HDs. CD8+ expansion differences were significant on day 14 under stimulation with IL-7/IL-15 [20-fold (HDs) vs. 7-fold expansion (patients) (*p* = 0.002)]. As for CD4+ CART_N_ cells, they showed significantly higher proliferation values from day 10 until the end of the culture under stimulation with IL-7/IL-15 [day 10: 7-fold (HDs) vs. 2-fold (patients) expansion (*p* = 0.0005), day 14: 42-fold (HDs) vs. 3-fold (patients) expansion (*p* = 0.0007), day 17: 77-fold (HDs) vs. 6-fold (patients) expansion (*p* = 0.004), and day 20: 62-fold (HDs) vs. 8-fold (patients) expansion (*p* = 0.005)].

Although the effect under stimulation with IL-7/IL-15 was marked, stimulation with IL-2 also led to a higher proliferation rate of CD4+ and CD8+ CART_N_ cells from HDs in comparison to untreated CLL patients (day 20: CD4 + CART_N_: 10-fold expansion; CD8+ CART_N_: 36-fold expansion). CD4+ CART_N_ cells of untreated patients, in turn, were not stimulated at all and even decreased with a net amplification of <1, whereas CD8+ CART_N_ cells showed a higher amplification rate (day 20: 23-fold expansion). Overall, the CD4+ naive CART expansion rate for HDs stimulated with IL-7/IL-15 on day 14 was significantly higher in comparison to IL-2 (*p* = 0.008).

### Detailed Phenotypic Analysis: IL-7/IL-15 vs. IL-2

**Figure 4:**
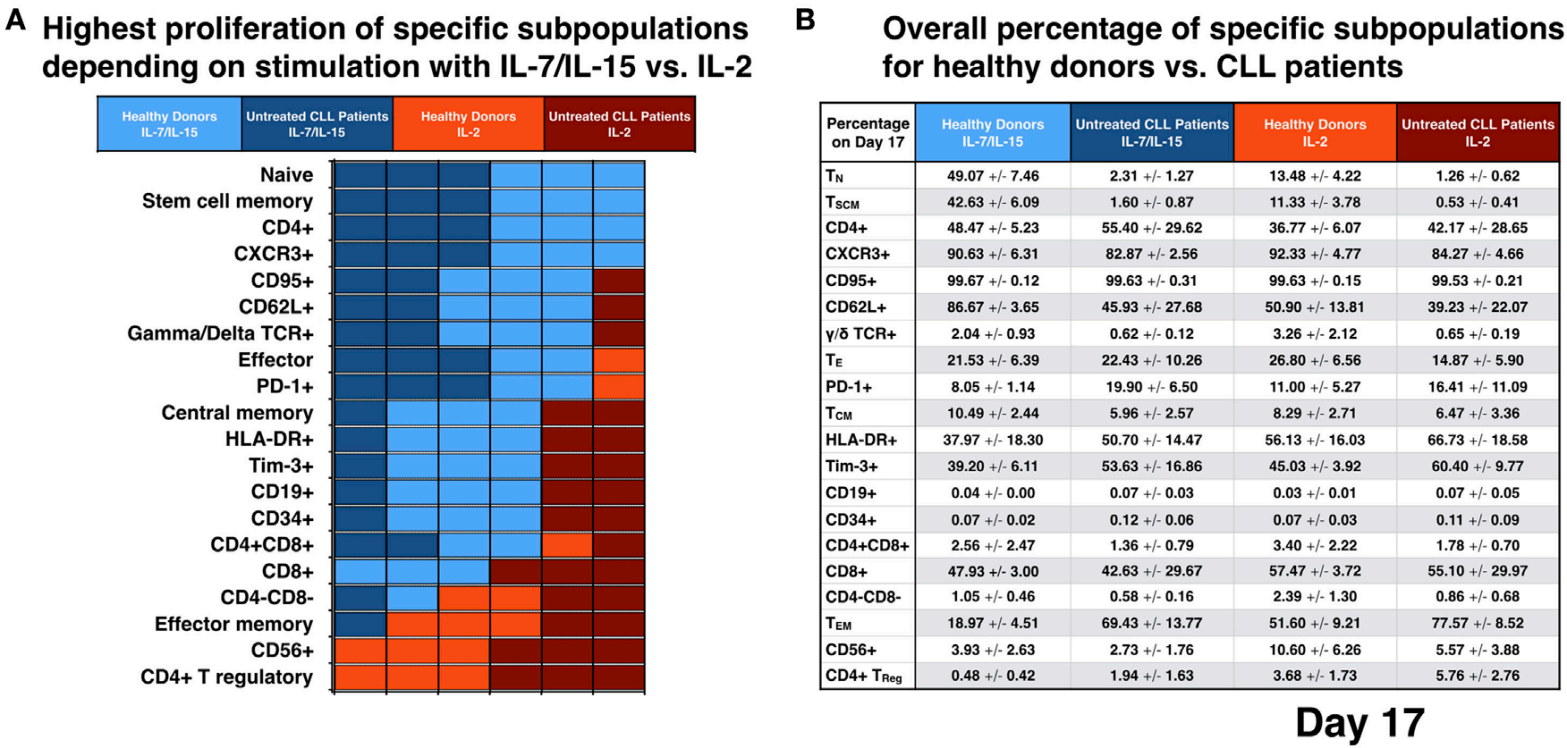
Analysis for different subpopulations of chimeric antigen receptor T (CART) cells from healthy donors (HDs) vs. patients on day 17 of culture with interleukin (IL)-7/IL-15 vs. IL-2. **(A)** The absolute cell number of specific cell subpopulations after stimulation with IL-7/IL-15 vs. IL-2 was compared for each donor independently (*n* = 6). Each unit on the x-axis represents one donor. Fields are highlighted with blue or red color according to the highest cell number of CART cells for each subtype, i.e., blue when proliferation resulted in higher absolute cell numbers under culture conditions with IL-7/IL-15 and red when proliferation resulted in higher absolute cell numbers under culture conditions with IL-2. Light colors were used for HDs, dark colors for patients. **(B)** The mean values of the percentage of CART cells with a specific phenotype on day 17 are represented and grouped into four categories: blue for stimulation with IL-7/IL-15, red for stimulation with IL-2 (HDs: light blue/red; patients: dark blue/red).

We analyzed which CART phenotype was preferentially stimulated by IL-7/IL-15 vs. IL-2 on day 17. Out of the six donors tested, it was evaluated how many presented a higher absolute cell number for a specific marker under stimulation with the two cytokine cocktails (distribution for a total of 20 CART subtypes depicted in Figure 4A; the mean percentages of CART cells of each subgroup for HDs and untreated CLL patients is illustrated in Figure 4B).

Impact of IL-7/IL-15: six out of six donors showed higher absolute T_N_, stem cell memory (T_SCM_), CD4+ and CXCR3+ CART cell numbers compared to generation in IL-2. T_SCM_ represent a subset of naive T cells and are characterized by the expression of CD27 and CD95 (27, 28). 87% of T_N_ cells in HDs and 69% in untreated CLL patients were T_SCM_ cells.

Five out of six donors (three HDs and two untreated CLL patients) had increased numbers of CART cells expressing CD95, CD62L, and γ/δ TCR and five out of six (two HDs and three untreated CLL patients) displayed a high absolute cell number of T_E_ and PD-1+ CART cells. Four out of six donors (three HDs and one untreated CLL patient) had higher levels of T_CM_, HLA-DR+, Tim-3+, CD19+, and CD34+ CART cells and four out of six (two HDs and two patients) presented a higher number of CD4+ CD8+ CART cells. However, for both the CD19 and the CD34 markers, the percentage of positive CART cells was overall very low for both cytokine cocktails (expressed on levels <0.2%).

The three HDs presented a higher absolute cell number of CD8+ CART cells after stimulation with IL-7/IL-15, while the three untreated CLL patients had higher levels of CD8+ CART cells after stimulation with IL-2.

Impact of IL-2: six out of six donors (three HDs and three untreated CLL patients) displayed an increased proportion of CD4+ T_Reg_ and CD56+ CART cells, five out of six (three HDs and two untreated CLL patients) presented a higher absolute cell number of T_EM_ CART cells and four out of six donors (two HDs and two untreated CLL patients) had an increased proportion of CD4−CD8− cells within their CART product compared to generation in IL-7/IL-15.

### Functional Evaluation of CART Cells

**Figure 5:**
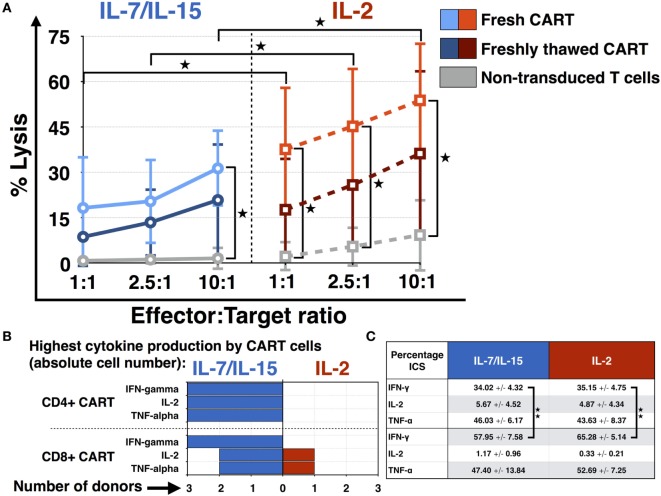
Functional characterization of chimeric antigen receptor T (CART) cells: cytotoxicity and cytokine expression. **(A)** The cytotoxic efficacy of CART cells against CD19+ Daudi cells (fresh *n* = 3, freshly thawed *n* = 3, non-transduced *n* = 3) was assessed after stimulation with interleukin (IL)-7/IL-15 vs. IL-2. The average lysis of CD19+ Daudi cells for different effector:target ratios (10:1, 5:1, 1:1) was determined by release of radioactive chromium-51 from labeled target cells. **(B)** Differences in the absolute cell number of CART cells expressing TNF-α, IFN-γ, and IL-2 after stimulation with IL-7/IL-15 vs. IL-2 are represented for each donor (*n* = 3). Fields are highlighted with blue or red color according to the highest percentage of CART cells for each subtype, i.e., blue for IL-7/IL-15 and red for IL-2. **(C)** The mean values of the percentage of CART cells with a specific phenotype on day 15 are represented (blue for stimulation with IL-7/IL-15, *n* = 3; red for stimulation with IL-2, *n* = 3). Significance is represented as * for *p* < 0.05 and ** for *p* < 0.01.

The cytotoxic efficacy of CART cells as well as non-transduced T cells was measured *via* chromium (Cr-51) release assay. Daudi cells were chosen as CD19+ target cell line and fresh CART cells were compared to freshly thawed CART cells. Additionally, the difference in lytic activity of CART cells stimulated with IL-7/IL-15 vs. IL-2 was assessed and is represented in Figure 5A. The effector:target ratios were 1:1, 2.5:1 as well as 10:1 and the number of viable CART cells was adjusted to the respective ratio. Fresh CART cells showed the highest cytotoxic efficacy and reached significance for both IL-7/IL-15 and IL-2 at the 10:1 ratio in comparison to non-transduced cells. At the 10:1 ratio, average lysis by fresh CART cells under stimulation with IL-7/IL-15 was 31 ± 13% (non-transduced T cells: 2 ± 4%, *p* = 0.017), whereas stimulation with IL-2 mediated lysis of 54 ± 19% (non-transduced T cells: 9 ± 12%, *p* = 0.026). Freshly thawed CART cells were less effective than fresh cells and showed a lytic activity of 21 ± 19 and 36 ± 27% at the 10:1 ratio, for stimulation with IL-7/IL-15 vs. IL-2, respectively. Moreover, the significantly higher lysis for fresh CART cells stimulated with IL-2 in comparison to those stimulated with IL-7/IL-15 was observable at every effector:target ratio (1:1 ratio: *p* = 0.028, 2.5:1 ratio: *p* = 0.024, 10:1 ratio: *p* = 0.026).

Figure 5B displays the ability of IL-7/IL-15 and IL-2 to induce cytokine-expressing CART cells on day 15 of culture. The absolute cell numbers were compared. IL-7/IL-15 led to a higher population of IFN-γ, IL-2, and TNF-α producing CD4+ as well as IFN-γ releasing CD8+ CART cells in three out of three donors (cells derived from one HD, two CLL patients) compared to IL-2. For IL-2 and TNF-α producing CD8+ CART cells, one donor displayed higher levels under stimulation with IL-2. Figure 5C displays the mean percentage of CART cells expressing IFN-γ, IL-2, and TNF-α on day 15. Significantly more CD8+ CART cells were expressing IFN-γ when compared to CD4+ cells. This higher cytokine production was observable for both IL-7/IL-15 (*p* = 0.009) and IL-2 stimulation (*p* = 0.002).

### Suboptimal Expansion of Naive CART Cells from Untreated CLL Patients

**Figure 6:**
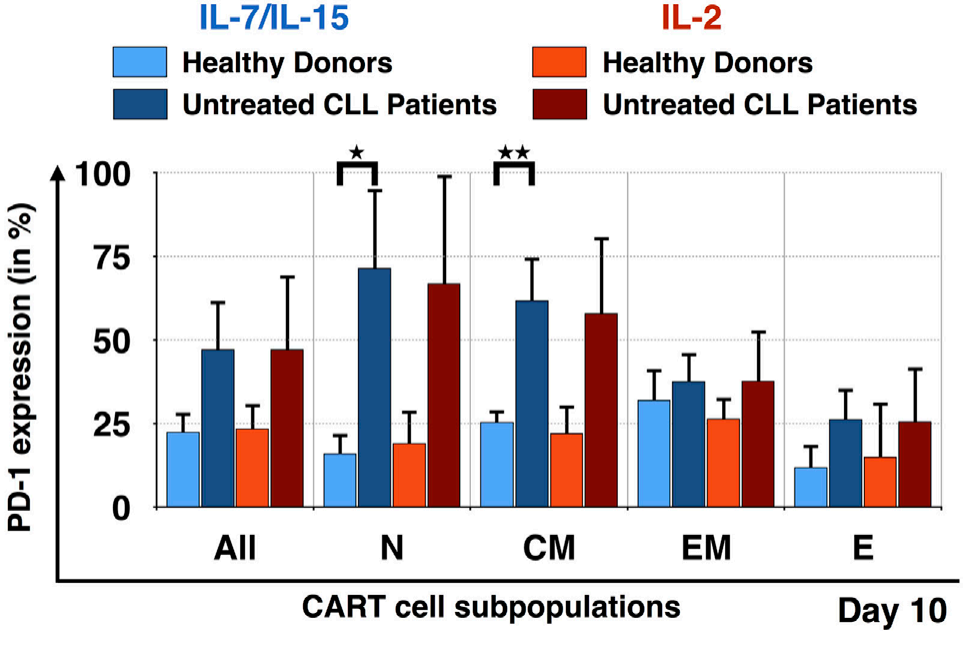
Exhaustion of naive T cell subpopulation for untreated chronic lymphocytic leukemia (CLL) patients in comparison to healthy donors (HDs). PD-1 expression on the surface of all chimeric antigen receptor T cells and the subpopulations T_N_, T_CM_, T_EM_, and T_E_ on day 10, for HDs (*n* = 3, light colors) and untreated CLL patients (*n* = 3, dark colors). The exhaustion marker expression was also compared according to the mode of stimulation, either with interleukin (IL)-7/IL-15 (blue) or with IL-2 (red). Significance is represented as * for *p* < 0.05 and ** for *p* < 0.01.

In order to better understand why it was difficult to expand CART_N_ cells derived from PBMC samples from untreated CLL patients, we evaluated the expression of exhaustion markers on CART cells during culture. Figure 6 illustrates the expression of the exhaustion marker PD-1 by the CART subpopulations at day 10. Samples from HDs stimulated with IL-7/IL-15 and IL-2 as well as samples from untreated CLL patients also stimulated with both cytokine cocktails were compared. Overall, CART cells derived from untreated patients displayed a higher percentage of PD-1 on their cell surface when compared to HDs [IL-7/IL-15: 23 ± 6% PD-1+ CART cells (HDs) vs. 47 ± 15% (untreated CLL patients); IL-2: 23 ± 7% PD-1+ CART cells (HDs) vs. 47 ± 22% (untreated CLL patients)]. The highest expression of PD-1 was detected on T_N_ [71 ± 24% of exhausted cells (patients) vs. 16 ± 6% (HDs) (*p* = 0.018)] and T_CM_ [62 ± 13% of exhausted cells (patients) vs. 25 ± 3% (HDs) (*p* = 0.009)] CART cells stimulated with IL-7/IL-15. With progressing differentiation, the level of exhaustion decreases, e.g., in CART_EM_ and T_E_ cells.

**Figure 7:**
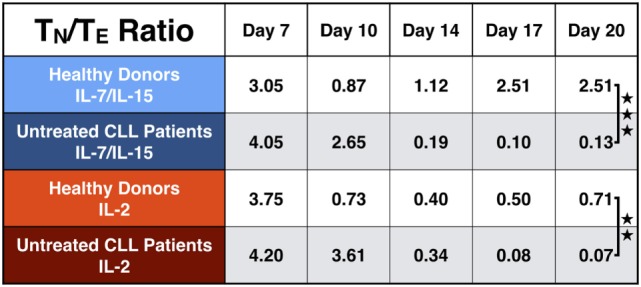
Naive to effector chimeric antigen receptor T (CART) ratio (T_N_/T_E_) during culture. Representation of the T_N_/T_E_ ratio for CART cells from healthy donors (*n* = 3, light colors) vs. untreated chronic lymphocytic leukemia patients (*n* = 3, dark colors) during culture. Stimulation with interleukin (IL)-7/IL-15 is marked in blue, stimulation with IL-2 in red. Significance is represented as ** for *p* < 0.01 and *** for *p* < 0.001.

As a high percentage of T_N_ cells within the final CART product correlates with long-lasting remissions in patients, we calculated the T_N_/T_E_ ratio from HDs and untreated CLL patients. The results are depicted in Figure 7. The T_N_/T_E_ ratio of HDs vs. untreated CLL patients became significant at the end of culture: on day 20, the T_N_/T_E_ ratio for HDs stimulated with IL-7/IL-15 reached the value 2.51. In contrast, untreated CLL patients showed a ratio of 0.13 (*p* = 0.0007). This effect was also visible when CART cells were stimulated with IL-2 (*p* = 0.004), although IL-2 was not able to promote expansion of CART_N_ cells as much as IL-7/IL-15.

## Discussion

Chimeric antigen receptor T cells have been designated as major breakthrough in cellular therapy for patients with cancer, particularly for patients with relapsed or refractory hematologic malignancies such as CD19+ leukemia and lymphoma. Hitherto, research has focused on genetic engineering in terms of vector design and costimulatory molecules. Only recently the “point of departure”, i.e., the characterization of PBMCs to be transduced, gained attention. Moreover, the influence of different cytokine cocktails on CART cultures has not yet been elucidated in depth. By analyzing the characteristics of HD cells that responded particularly well to cytokine stimuli and comparing CART products generated from HDs and untreated CLL patients under similar conditions, this study aimed to define the contribution of cytokine stimulation and culture conditions to resultant phenotype of CART products.

Since response failure in clinical CART therapy has been associated with an insufficient number of T_N_ cells within the CART cells (25), we screened PBMCs from HDs and untreated CLL patients for percentage of T_N_ cells. In comparison with HDs, CLL patients contained significantly lower T_N_ cells within their PBMCs.

Following screening, HDs and CLL patients displaying comparable levels of T_N_ cells were chosen for CART production. CART cells were successfully generated for all included individuals with similar transduction efficiencies under two stimulating cytokine conditions, i.e., IL-7/IL-15 and IL-2. When comparing the expansion of the subpopulations T_N_, T_CM_, T_EM_, and T_E_ during CART culture, significant differences were observed under distinct culture conditions: IL-7/IL-15 cytokine stimulation led to an increase of T_N_, T_SCM_, CD4+, CXCR3+, CD62L+, γ/δ TCR+, T_E_, PD-1+, T_CM_, HLA-DR+, Tim-3+, and CD4+ CD8+ CART cells, whereas an IL-2 culture condition stimulated T_EM_, CD56+, immunosuppressive CD4−CD8− and CD4+ T_Reg_ CART cells.

**Figure 8:**
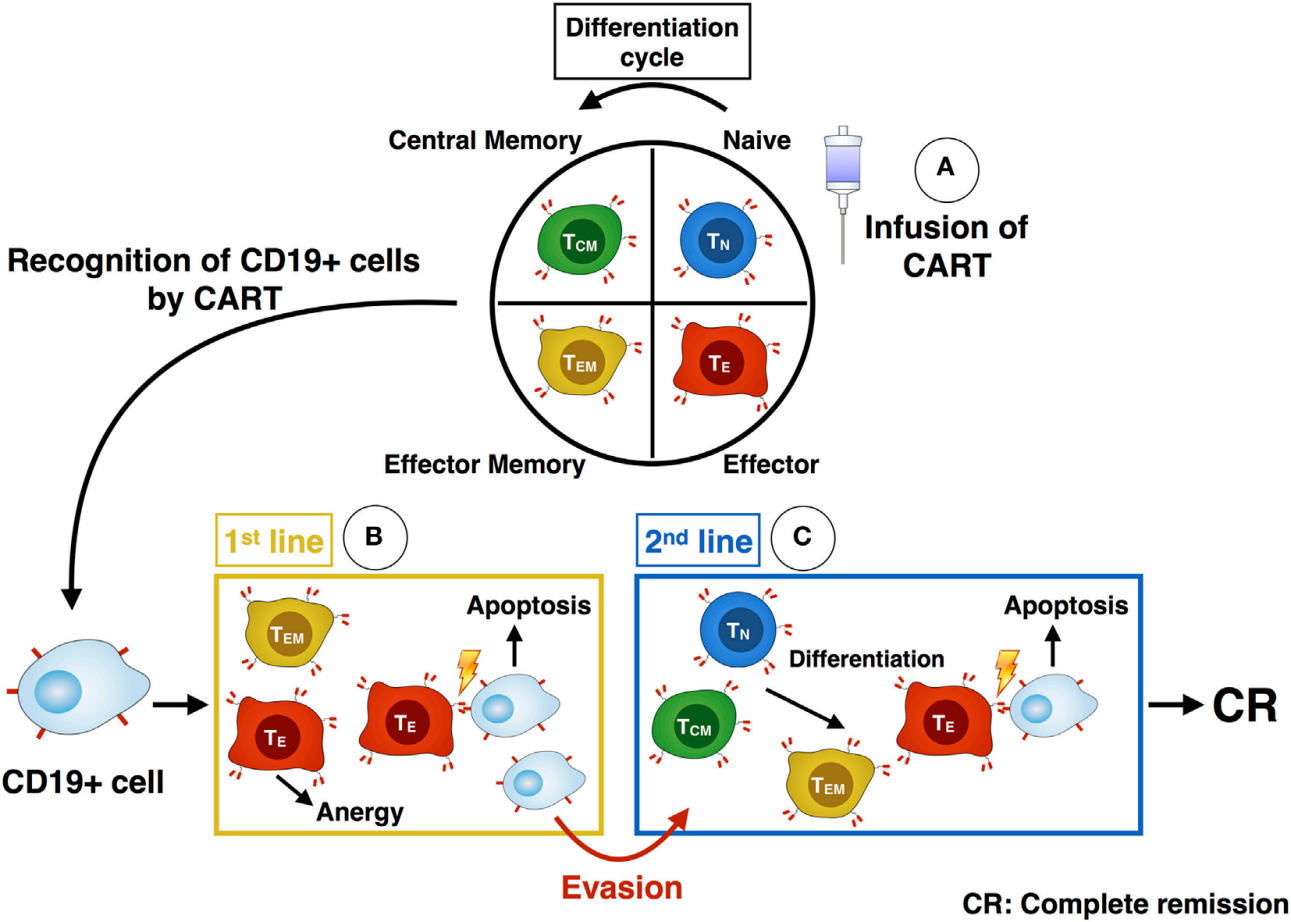
Illustration of the differentiation and killing process of chimeric antigen receptor T (CART) subpopulations. After administration of CART cells into the patient **(A)**, the more differentiated T_EM_ and T_E_ CART cells are responsible for the first killing of CD19+ cells **(B)**. As they become anergic, the less-differentiated T_N_ and T_CM_ subsets build up a second line of effector CART cells **(C)**. They differentiate into T_EM_ and T_E_ and are able to kill remaining malignant cells, which evaded the immune defense beforehand. Ideally, these cells eventually mediate a complete response within the patient.

The functional evaluation *via* chromium release assay showed a higher cytotoxic activity of CART cells against CD19+ target cells after stimulation with IL-2 compared to stimulation with IL-7/IL-15. The higher cytotoxic efficacy of IL-2 stimulated CART cells can be explained given the higher proportion of differentiated effector CART cells (T_EM_ and CD56+) specialized in killing. In contrast, stimulation with IL-7/IL-15 mediated a higher proportion of immature subtypes (T_N_, T_SCM_) within the CART product, thus leading to lower overall lytic capacity. *In vivo* killing of malignant cells by CART is more complex and depends on the different CART subpopulations acting over time (Figure 8): the first attack after infusion of CART cells into the patient is mediated by highly differentiated cells endowed with high lytic activity. However, these cells soon reach the end of differentiation and become anergic. Without a second line of attacking immune cells, patients would face relapse after loss of effector cells. Long-lasting T_N_ or T_CM_ CART subtypes with self-renewal capacity can act as a reservoir for a second line of effector CART cells (18, 19). Therefore, infusing a high percentage of CART_N_ cells into patients is highly desirable and represents a source for new effector cells over time, eventually contributing to higher survival rate of treated patients. Consequently, a high percentage of T_N_ cells at the beginning of the CART culture correlates with *in vivo* CART expansion (29).

We expanded CART_N_ cells the most successfully in samples derived from HDs under stimulation with IL-7/IL-15. However, similar expansion rates of CART_N_ cells could not be achieved for samples derived from untreated CLL patients, independent of the addition of IL-7/IL-15 or IL-2. As a consequence, T_N_/T_E_ ratio on day 20 of culture was high for HDs, but very low for CART cells derived from untreated patients.

The main reason for this might be the cellular composition of the starting material: whereas other parameters were similar between the tested individuals from both groups, untreated CLL patients showed a high percentage of malignant B cells contained within their PBMCs. These were cultured together with the CD3+ T cells, but received no activation signal through anti-CD3/anti-CD28 antibodies and underwent apoptosis. In fact, on day 17 of culturing B cells had vanished (percentage of CD19+ cells < 0.01), resulting in a culture made up solely of CART and non-transduced T cells.

As reported by Gattinoni et al. for an *in vivo* model, endogenous cells compete with transferred T cells for supportive cytokines IL-7/IL-15 (30). A similar effect might have occurred during our CART culture: B cells in samples from untreated CLL patients might have taken up most of the added cytokines, thus restricting the availability for CART cells. As a consequence, CART_N_ expansion normally promoted by IL-7/IL-15 was disturbed. Lacking cytokine stimulation induced the exhaustive phenotype expressing high levels of PD-1 observed on day 10 of culture of the less-differentiated CART cells, i.e., T_N_ and T_CM_. Moreover, given the (1) high frequency of CLL cells present during the early culture period and (2) the known contribution of the microenvironment on CART cell generation, it is possible that CLL cells within the culture are recognized by the CART cells leading to differentiation from naive to effector cells. As the frequency of B cells within samples from HDs is significantly lower, this effect is less prominent in HD culture conditions.

In clinical studies, lymphodepletion is performed through a conditioning cytostatic regimen preceding CART cell infusion in order to make room for CART expansion and enhance the homeostatic microenvironment. This has been identified as a crucial factor for CART cell engraftment (31–34). However, patient samples used to generate the CART product are taken before lymphodepletion and the peripheral blood of patients might still contain high numbers of malignant B cells, interfering with the expansion of naive T cells. As a consequence, our data support the selection of T cells either by magnetic-activated cell sorting or by flow cytometry to obtain pure T cells and enable efficient expansion of CART_N_ cells *ex vivo*.

In conclusion, we aimed to establish a preclinical potency assay for CART generation. A high percentage of less-differentiated T_N_ cells contributes to a long-lasting remission and significant expansion of CART cells *in vivo* (25, 35–40). According to our results, these conditions are fulfilled when a cocktail of IL-7 and IL-15 is used instead of IL-2 as stimulating cytokines. High expansion of long-lasting CART_N_ cells was achieved when PBMCs from HDs were used. In contrast, untreated CLL patients remain a particular challenge for production of CART cells targeting the CD19 antigen, as clinical responses and persistence *in vivo* is limited in CART products with low proportions of T_N_. High amounts of B cells in untreated CLL patients might be responsible for this. Hence B cell depletion before starting CART production might constitute a tool to overcome this problem. As CART cells emerge as frontline therapy, manufacture may be hindered in untreated CLL patients due to high frequencies of B cells in the initial culture. Our data therefore support either B cell depletion or T cell sorting early on, which could allow more T_N_ to expand. In line with others (41–44), B cell depletion in hematologic malignancies also before infusing CART cells is advisable. Subsequent analyses of the PBMC content factors that determine CART efficacy might further improve the results of CART cell therapy. However, the additional impact of differences in CAR construct, target antigen, manufacture protocol and patient disease status also likely play a role. *In vitro* studies with larger cohorts of CLL patients vs. HDs as well as evaluation of clinical samples from patients treated with CD19.CART cells are ongoing and might result in future selection or purging strategies.

## Ethics Statement

The manuscript contains data on patient samples. This study was carried out in accordance with the recommendations of the Institutional Review Board (IRB) of the Heidelberg University Hospital with written informed consent from all subjects. All subjects gave written informed consent in accordance with the Declaration of Helsinki. The protocol (S-254/2016) was approved by the IRB of the Heidelberg University Hospital.

## Author Contributions

MS designed the study; J-MH performed the experiments and analyzed the data; J-MH, M-LS, and MS wrote the manuscript; LW, SS, AH, CK, and AS discussed experimental design; LS, CK, PW, SH, AH, CM-T, and PD reviewed the manuscript; UG and AL set up the CART culture protocol and reviewed the manuscript.

## Conflict of Interest Statement

The authors have no relevant affiliations or financial involvement with any organization or entity with a financial interest in or financial conflict with the subject matter or materials discussed in the manuscript. This includes employment, consultancies, honoraria, stock ownership or options, expert testimony, grants or patents received or pending, or royalties. The reviewers RM and MD and handling editor declared their shared affiliation.

## Supplementary Material

Figure S1 FACS data. Illustration of the gating strategy used during acquisition with the LSRII flow cytometer. The first gate assessed viable cells, the second gate CD3+ cells and the third gate CD3+ CART cells. Out of the CD3+ CART cells, the percentages of CD4+/CD8+ cells were evaluated. T_N_, T_CM_, T_EM_, and T_E_ subpopulations were defined and expression of the exhaustion marker programmed cell death 1 was assessed. As representative examples data from HD 7 and untreated chronic lymphocytic leukemia patient 2 (P 2) at day 17 of culture after stimulation with interleukin (IL)-7/IL-15 are displayed here.

## References

[1] Dotti G Gottschalk S Savoldo B Brenner MK. Design and development of therapies using chimeric antigen receptor-expressing T cells. Immunol Rev (2014) 257(1):107–26. 10.1111/imr.12131 24329793 PMC3874724

[2] Ramos CA Heslop HE Brenner MK. CAR-T cell therapy for lymphoma. Annu Rev Med (2016) 67:165–83. 10.1146/annurev-med-051914-021702 26332003 PMC4732525

[3] Ramos CA Savoldo B Dotti G CD19-CAR trials. Cancer J (2014) 20(2):112–8. 10.1097/PPO.0000000000000031 24667955 PMC3979594

[4] Park JH Geyer MB Brentjens RJ. CD19-targeted CAR T-cell therapeutics for hematologic malignancies: interpreting clinical outcomes to date. Blood (2016) 127(26):3312–20. 10.1182/blood-2016-02-629063 27207800 PMC4929923

[5] Schubert ML Huckelhoven A Hoffmann JM Schmitt A Wuchter P Sellner L Chimeric antigen receptor T cell therapy targeting CD19-positive leukemia and lymphoma in the context of stem cell transplantation. Hum Gene Ther (2016) 27(10):758–71. 10.1089/hum.2016.097 27479233

[6] Geyer MB Brentjens RJ. Review: current clinical applications of chimeric antigen receptor (CAR) modified T cells. Cytotherapy (2016) 18(11):1393–409. 10.1016/j.jcyt.2016.07.003 27592405 PMC5067198

[7] Lee DW Kochenderfer JN Stetler-Stevenson M Cui YK Delbrook C Feldman SA T cells expressing CD19 chimeric antigen receptors for acute lymphoblastic leukaemia in children and young adults: a phase 1 dose-escalation trial. Lancet (2015) 385(9967):517–28. 10.1016/S0140-6736(14)61403-3 25319501 PMC7065359

[8] Weiland J Elder A Forster V Heidenreich O Koschmieder S Vormoor J. CD19: a multifunctional immunological target molecule and its implications for Blineage acute lymphoblastic leukemia. Pediatr Blood Cancer (2015) 62(7):1144–8. 10.1002/pbc.25462 25755168

[9] Sadelain M. CAR therapy: the CD19 paradigm. J Clin Invest (2015) 125(9):3392–400. 10.1172/JCI80010 26325036 PMC4588281

[10] Loskog A Giandomenico V Rossig C Pule M Dotti G Brenner MK. Addition of the CD28 signaling domain to chimeric T-cell receptors enhances chimeric T-cell resistance to T regulatory cells. Leukemia (2006) 20(10):1819–28. 10.1038/sj.leu.2404366 16932339

[11] Savoldo B Ramos CA Liu E Mims MP Keating MJ Carrum G CD28 costimulation improves expansion and persistence of chimeric antigen receptor-modified T cells in lymphoma patients. J Clin Invest (2011) 121(5):1822–6. 10.1172/JCI46110 21540550 PMC3083795

[12] Long AH Haso WM Shern JF Wanhainen KM Murgai M Ingaramo M 4-1BB costimulation ameliorates T cell exhaustion induced by tonic signaling of chimeric antigen receptors. Nat Med (2015) 21(6):581–90. 10.1038/nm.3838 25939063 PMC4458184

[13] Karlsson H Svensson E Gigg C Jarvius M Olsson-Stromberg U Savoldo B Evaluation of intracellular signaling downstream chimeric antigen receptors. PLoS One (2015) 10(12):e0144787. 10.1371/journal.pone.0144787 26700307 PMC4689545

[14] Porter DL Hwang WT Frey NV Lacey SF Shaw PA Loren AW Chimeric antigen receptor T cells persist and induce sustained remissions in relapsed refractory chronic lymphocytic leukemia. Sci Transl Med (2015) 7(303):303ra139. 10.1126/scitranslmed.aac5415 26333935 PMC5909068

[15] Maus MV June CH. Making better chimeric antigen receptors for adoptive T-cell therapy. Clin Cancer Res (2016) 22(8):1875–84. 10.1158/1078-0432.CCR-15-1433 27084741 PMC4843171

[16] Rosenberg SA Yang JC Sherry RM Kammula US Hughes MS Phan GQ Durable complete responses in heavily pretreated patients with metastatic melanoma using T-cell transfer immunotherapy. Clin Cancer Res (2011) 17(13):4550–7. 10.1158/1078-0432.CCR-11-0116 21498393 PMC3131487

[17] Enblad G Karlsson H Loskog AS. CAR T-cell therapy: the role of physical barriers and immunosuppression in lymphoma. Hum Gene Ther (2015) 26(8):498–505. 10.1089/hum.2015.054 26230974 PMC4554546

[18] Sommermeyer D Hudecek M Kosasih PL Gogishvili T Maloney DG Turtle CJ Chimeric antigen receptor-modified T cells derived from defined CD8+ and CD4+ subsets confer superior antitumor reactivity in vivo. Leukemia (2016) 30(2):492–500. 10.1038/leu.2015.247 26369987 PMC4746098

[19] Gattinoni L Klebanoff CA Restifo NP. Paths to stemness: building the ultimate antitumour T cell. Nat Rev Cancer (2012) 12(10):671–84. 10.1038/nrc3322 22996603 PMC6352980

[20] Farber DL Yudanin NA Restifo NP. Human memory T cells: generation, compartmentalization and homeostasis. Nat Rev Immunol (2014) 14(1):24–35. 10.1038/nri3567 24336101 PMC4032067

[21] Busch DH Frassle SP Sommermeyer D Buchholz VR Riddell SR. Role of memory T cell subsets for adoptive immunotherapy. Semin Immunol (2016) 28(1):28–34. 10.1016/j.smim.2016.02.001 26976826 PMC5027130

[22] Rathmell JC Farkash EA Gao W Thompson CB. IL-7 enhances the survival and maintains the size of naive T cells. J Immunol (2001) 167(12):6869–76. 10.4049/jimmunol.167.12.6869 11739504

[23] Berard M Brandt K Bulfone-Paus S Tough DF IL-15 promotes the survival of naive and memory phenotype CD8+ T cells. J Immunol (2003) 170(10):5018–26. 10.4049/jimmunol.170.10.5018 12734346

[24] Cieri N Camisa B Cocchiarella F Forcato M Oliveira G Provasi E IL-7 and IL-15 instruct the generation of human memory stem T cells from naive precursors. Blood (2013) 121(4):573–84. 10.1182/blood-2012-05-431718 23160470

[25] Xu Y Zhang M Ramos CA Durett A Liu E Dakhova O Closely related T-memory stem cells correlate with in vivo expansion of CAR.CD19-T cells and are preserved by IL-7 and IL-15. Blood (2014) 123(24):3750–9. 10.1182/blood-2014-01-552174 24782509 PMC4055922

[26] Schmitt M Schmitt A Rojewski MT Chen J Giannopoulos K Fei F RHAMM-R3 peptide vaccination in patients with acute myeloid leukemia, myelodysplastic syndrome, and multiple myeloma elicits immunologic and clinical responses. Blood (2008) 111(3):1357–65. 10.1182/blood-2007-07-099366 17978170

[27] Gattinoni L Lugli E Ji Y Pos Z Paulos CM Quigley MF A human memory T cell subset with stem cell-like properties. Nat Med (2011) 17(10):1290–7. 10.1038/nm.2446 21926977 PMC3192229

[28] Hendriks J Gravestein LA Tesselaar K van Lier RA Schumacher TN Borst J. CD27 is required for generation and long-term maintenance of T cell immunity. Nat Immunol (2000) 1(5):433–40. 10.1038/80877 11062504

[29] Klaver Y van Steenbergen SC Sleijfer S Debets R Lamers CH. T cell maturation stage prior to and during GMP processing informs on CAR T cell expansion in patients. Front Immunol (2016) 7:648. 10.3389/fimmu.2016.00648 28082983 PMC5183620

[30] Gattinoni L Finkelstein SE Klebanoff CA Antony PA Palmer DC Spiess PJ Removal of homeostatic cytokine sinks by lymphodepletion enhances the efficacy of adoptively transferred tumor-specific CD8+ T cells. J Exp Med (2005) 202(7):907–12. 10.1084/jem.20050732 16203864 PMC1397916

[31] Turtle CJ Hanafi LA Berger C Gooley TA Cherian S Hudecek M CD19 CAR-T cells of defined CD4+:CD8+ composition in adult B cell ALL patients. J Clin Invest (2016) 126(6):2123–38. 10.1172/JCI85309 27111235 PMC4887159

[32] Turtle CJ Hay KA Hanafi LA Li D Cherian S Chen X Durable molecular remissions in chronic lymphocytic leukemia treated with CD19-specific chimeric antigen receptor-modified T cells after failure of ibrutinib. J Clin Oncol (2017) 35(26):3010–20. 10.1200/JCO.2017.72.8519 28715249 PMC5590803

[33] Brentjens RJ Riviere I Park JH Davila ML Wang X Stefanski J Safety and persistence of adoptively transferred autologous CD19-targeted T cells in patients with relapsed or chemotherapy refractory B-cell leukemias. Blood (2011) 118(18):4817–28. 10.1182/blood-2011-04-348540 21849486 PMC3208293

[34] Zhang T Cao L Xie J Shi N Zhang Z Luo Z Efficiency of CD19 chimeric antigen receptor-modified T cells for treatment of B cell malignancies in phase I clinical trials: a meta-analysis. Oncotarget (2015) 6(32):33961–71. 10.18632/oncotarget.5582 26376680 PMC4741817

[35] Barrett DM Singh N Liu X Jiang S June CH Grupp SA Relation of clinical culture method to T-cell memory status and efficacy in xenograft models of adoptive immunotherapy. Cytotherapy (2014) 16(5):619–30. 10.1016/j.jcyt.2013.10.013 24439255 PMC3988256

[36] Gargett T Brown MP. Different cytokine and stimulation conditions influence the expansion and immune phenotype of third-generation chimeric antigen receptor T cells specific for tumor antigen GD2. Cytotherapy (2015) 17(4):487–95. 10.1016/j.jcyt.2014.12.002 25573334

[37] Xu XJ Song DG Poussin M Ye Q Sharma P Rodriguez-Garcia A Multiparameter comparative analysis reveals differential impacts of various cytokines on CART cell phenotype and function ex vivo and in vivo. Oncotarget (2016) 7(50):82354–68. 10.18632/oncotarget.10510 27409425 PMC5347696

[38] Yang S Ji Y Gattinoni L Zhang L Yu Z Restifo NP Modulating the differentiation status of ex vivo-cultured anti-tumor T cells using cytokine cocktails. Cancer Immunol Immunother (2013) 62(4):727–36. 10.1007/s00262-012-1378-2 23207483 PMC6354242

[39] Klebanoff CA Gattinoni L Restifo NP. Sorting through subsets: which T-cell populations mediate highly effective adoptive immunotherapy? J Immunother (2012) 35(9):651–60. 10.1097/CJI.0b013e31827806e6 23090074 PMC3501135

[40] Hinrichs CS Borman ZA Gattinoni L Yu Z Burns WR Huang J Human effector CD8+ T cells derived from naive rather than memory subsets possess superior traits for adoptive immunotherapy. Blood (2011) 117(3):808–14. 10.1182/blood-2010-05-286286 20971955 PMC3035075

[41] James SE Orgun NN Tedder TF Shlomchik MJ Jensen MC Lin Y Antibody-mediated B-cell depletion before adoptive immunotherapy with T cells expressing CD20-specific chimeric T-cell receptors facilitates eradication of leukemia in immunocompetent mice. Blood (2009) 114(27):5454–63. 10.1182/blood-2009-08-232967 19880489 PMC2798862

[42] Cheadle EJ Hawkins RE Batha H O’Neill AL Dovedi SJ Gilham DE Natural expression of the CD19 antigen impacts the long-term engraftment but not antitumor activity of CD19-specific engineered T cells. J Immunol (2010) 184(4):1885–96. 10.4049/jimmunol.0901440 20089697

[43] Feucht J Kayser S Gorodezki D Hamieh M Doring M Blaeschke F T-cell responses against CD19+ pediatric acute lymphoblastic leukemia mediated by bispecific T-cell engager (BiTE) are regulated contrarily by PD-L1 and CD80/CD86 on leukemic blasts. Oncotarget (2016) 7(47):76902–19. 10.18632/oncotarget.12357 27708227 PMC5363558

[44] Kono H Rock KL. How dying cells alert the immune system to danger. Nat Rev Immunol (2008) 8(4):279–89. 10.1038/nri2215 18340345 PMC2763408

