# Supplementary figures and images for: Differences in Expansion Potential of Naive Chimeric Antigen Receptor T Cells from Healthy Donors and Untreated Chronic Lymphocytic Leukemia Patients
Source: Front Immunol. 2018 Jan 10;8:1956. doi: 10.3389/fimmu.2017.01956 (PMC5767585; doi:10.3389/fimmu.2017.01956)

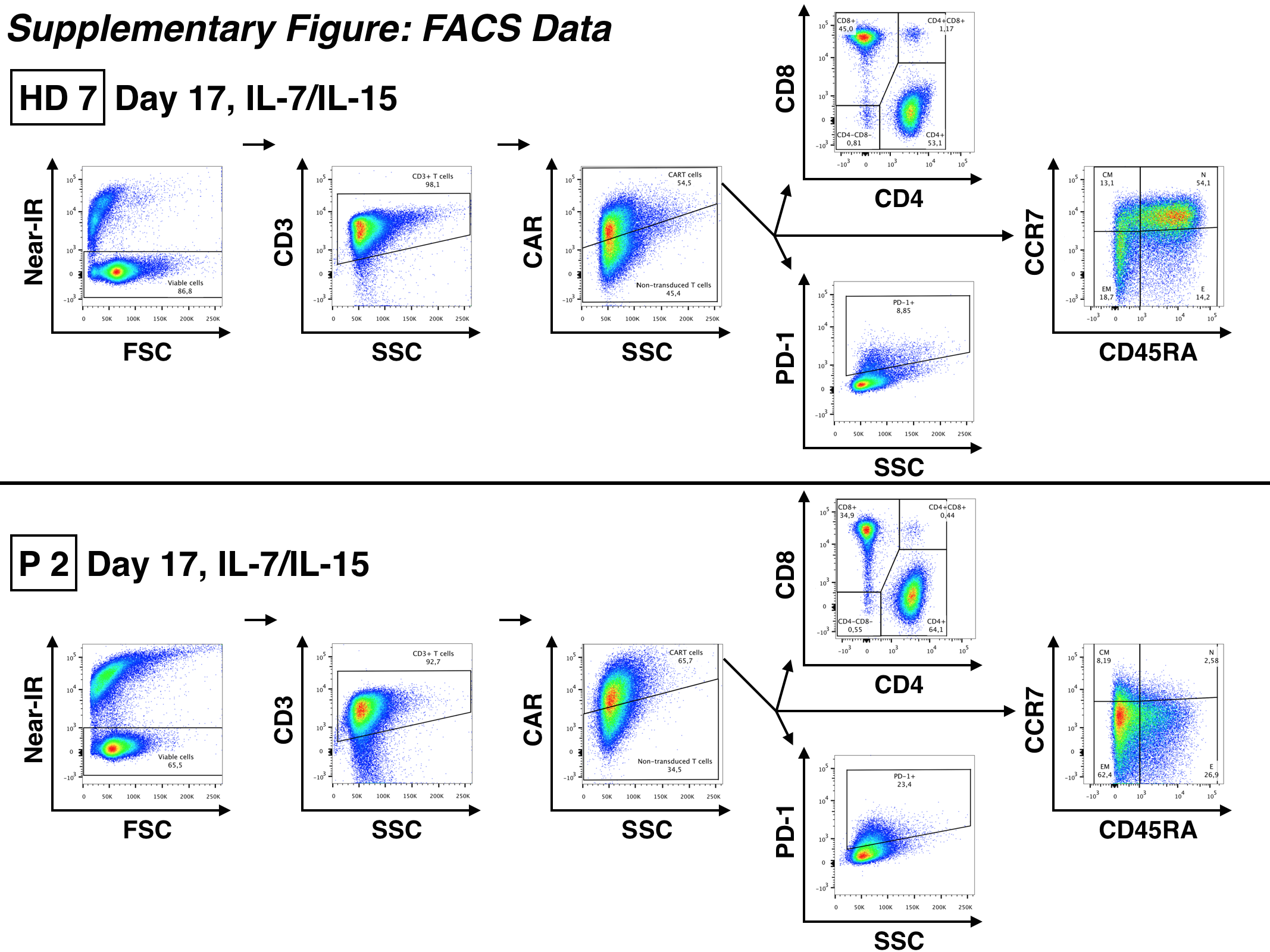

Supplement: Figure S1 — FACS data. Illustration of the gating strategy used during acquisition with the LSRII flow cytometer. The first gate assessed viable cells, the second gate CD3+ cells and the third gate CD3+ CART cells. Out of the CD3+ CART cells, the percentages of CD4+/CD8+ cells were evaluated. TN, TCM, TEM, and TE subpopulations were defined and expression of the exhaustion marker programmed cell death 1 was assessed. As representative examples data from HD 7 and untreated chronic lymphocytic leukemia patient 2 (P 2) at day 17 of culture after stimulation with interleukin (IL)-7/IL-15 are displayed here. [file image_1.png]
